# Rhegmatogenous Retinal Detachment Secondary to Type I Stickler Syndrome: Diagnosis, Treatment and Long-Term Outcomes

**DOI:** 10.3390/genes15111455

**Published:** 2024-11-11

**Authors:** Xin Chen, Yuqiao Ju, Fengjuan Gao, Yuan Zong, Ting Zhang, Ruiwen Li, Qing Chang, Xin Huang

**Affiliations:** 1 Eye Institute and Department of Ophthalmology, Eye & ENT Hospital, Fudan University, Shanghai 200031, China; 2 NHC Key Laboratory of Myopia and Related Eye Diseases, Key Laboratory of Myopia and Related Eye Diseases, Chinese Academy of Medical Sciences, Shanghai 200031, China; 3 Shanghai Key Laboratory of Visual Impairment and Restoration, Shanghai 200031, China

**Keywords:** rhegmatogenous retinal detachment, Stickler syndrome, pars plana vitrectomy, genetic testing, *COL2A1*

## Abstract

Objective: This study aimed to clarify the genetic diagnosis of rhegmatogenous retinal detachment (RRD) secondary to type I Stickler syndrome (STL1) and evaluate the anatomical and functional outcomes of surgical treatment. Methods: This retrospective study included 11 patients with RRD secondary to STL1. Familial and sporadic cases of STL1 were diagnosed at the Eye & ENT Hospital, Fudan University, between 2017 and 2023. To clarify the genetic diagnosis, next-generation sequencing was performed in suspected STL1 cases. Further, standard ocular examinations and surgical treatment were performed. Results: Nine variants of *COL2A1*, including four novel mutations (c.394G>T, c.2977G>T, c.3003+2dup, and c.3853G>C), were screened and identified. The pathogenicity of all variants was conclusively demonstrated. Among patients who underwent vitrectomy, the mean age at RRD was 11.5 years, and the mean follow-up was 32.9 months. The average number of surgical procedures required during the follow-up was two; 90.9% of eyes achieved final attachment, and best corrected visual acuity (BCVA) significantly improved in 81.8% of the eyes, with a middle postoperative logMAR BCVA of 0.52 compared with the preoperative value (*p* = 0.0148). High intraocular pressure (81.8%) and cataract (72.7%) were the most common complications. Conclusions: Our study expands the spectrum of *COL2A1* mutations and provides a novel diagnostic strategy for STL1. By combining clinical manifestations with genetic testing, STL1 could be accurately diagnosed. With proper surgical treatment and long-term follow-up, the prognosis of RRD in patients with STL1 could be improved.

## 1. Introduction

Stickler syndrome (STL) is a group of inherited connective tissue disorders involving the eye, inner ear, skeleton, and cartilage and was first described by Stickler [1] in 1965. The incidence of STL among newborns is relatively rare worldwide. It ranges from 1:7500 to 1:10,000 [2]. With the universalization of next-generation sequencing, STL has been divided into ten subgroups based on eight distinct genes responsible for the production of different collagen types [3]. The inheritance of STL is frequently autosomal dominant (AD), and autosomal recessive inheritance is rarely seen [3]. Type I STL (STL1) is the most common, accounting for approximately 80–90% of all STL cases, and is associated with the AD pathogenic gene *COL2A1* [4]. However, the clinical manifestations of STL1 are usually more heterogeneous, resulting in misdiagnosis or missed diagnosis, especially in patients presenting with mild or even a lack of systemic features at an early age [5,6]. By combining clinical characteristics and pathogenic variants, a definitive genetic diagnosis of STL1 could be possible. This can also prompt early follow-up of the inner ear and skeleton to improve prognosis with comprehensive treatments.

The most typical ocular manifestation of STL1 is high myopia, which generates vitreous liquid extremely early. These changes may lead to a high risk of rhegmatogenous retinal detachment (RRD), the most common threat to vision in patients with STL1. In a previous study, the incidence of RRD in patients with STL1 was as high as 49% throughout their life, while over one-third had bilateral RRD [7,8]. Owing to the frequent presence of giant retinal tears, late detection, and rapid progression, >20% of patients have inoperable RRD at presentation, and outcomes from surgical repair are comparatively poor, with primary success achieved in only 50–55% of cases [9,10]. Therefore, there is an urgent need to improve the effectiveness of treatment for RRD secondary to STL1.

In this study, we aimed to clarify the genetic diagnosis of suspected STL1 in patients with secondary RRD. We performed pars plana vitrectomy (PPV) in these patients and evaluated their anatomical and functional outcomes. Through this study, we aim to deepen our understanding of STL1 and draw clinicians’ attention toward the diagnosis and treatment of ocular genetic syndromes.

## 2. Materials and Methods

This retrospective study was approved by the Ethical Oversight Committee of Eye and ENT Hospital, Fudan University, and the approval number was [2020] (2020119). The study was conducted according to the tenets of the Declaration of Helsinki, and all the patients and their family members provided signed informed consent for this research.

This study was carried out between 2017 and 2023 at the Eye & ENT Hospital, Fudan University. Patients who had RRD with high myopia as the first presentation, in combination with other typical clinical manifestations, including midface hypoplasia, hearing loss and joint disabilities, were highly suspected of STL1. High myopia was defined as axial length (AL) ≥ 26.00 mm or refractive errors ≤ −6.0 diopter spherical equivalent. Genetic testing was performed in these families and in sporadic cases.

### 2.1. Ocular Examination

All patients underwent standard ophthalmologic examinations, including visual acuity (VA) tests, slit-lamp microscopy, B-ultrasonography, optical coherence tomography (OCT) (SS-OCT, Heidelberg Engineering, Heidelberg, Germany), and wide-field fundoscopy (Optos 200Tx, Optos, Marlborough, MA, USA). The ZEISS IOL Master 700^®^ device (Carl Zeiss Meditec, Inc., Jena, Germany) was used to measure preoperative biometric data, including the AL, flattest and steepest keratometry (K) values, anterior chamber depth (ACD), lens thickness (LT), and horizontal corneal diameter (CD). Best corrected visual acuity (BCVA) was assessed using a Snellen chart and converted into logMAR BCVA for statistical analysis. Foveal hypoplasia (FH) describes a condition in which the fovea has a characteristic morphologic abnormality associated with disruption of the normal embryological development of the retina [11]. Considering that there might be abnormal manifestations such as FH in contralateral eyes, OCT scan was used to assess the presence and grade of FH according to the Leicester Grading System [12].

### 2.2. Surgical Treatment

Ophthalmic treatments included retinal laser photocoagulation, cataract phacoemulsification, intraocular lens (IOL) implantation, PPV with C3F8 or silicone oil tamponade, and silicone oil removal (SOR). Dr. Xin Huang and Dr. Qing Chang performed all the surgeries. Three-port, 23- or 25-gauge pars plana vitrectomy with valved cannula was performed. After core vitrectomy, triamcinolone acetonide (TA) was used in cortex removal and 360° peripheral vitreous shaving visualized by scleral indentation was completed. Laser photocoagulation was performed to treat retinal lattice degeneration, tear(s), and break(s). Subretinal fluid was drained through the break(s) responsible for the retinal detachment. The use of perfluorooctane, C3F8, and silicone oil was at the surgeon’s discretion.

As part of treatment, cataract extraction and IOL implantation play important roles in patients’ final visual quality. There have been no previous studies on the choice of IOL power calculation formula in eyes with high myopia in STL1 cases with silicone oil tamponade. We compared several regular formulae, such as the Barrett Universal II, SRK/T, Holladay, Haigis, and Hoffer Q, with a novel machine-learning-based formula, the Zhu-Lu formula. The mean prediction error (△SD) was defined as actual postoperative refraction minus predicted refraction back-calculated, with the implanted IOL power using each formula. The mean absolute prediction error (|△SD|) and △SD were calculated and compared.

To compare the IOL power calculation formula, we recruited two more patients with STL1 who were family members of Patients 4 and 11 (Figure S1). They underwent RRD surgery at another hospital and complicated cataract surgery at our hospital.

### 2.3. Genetic Testing and Pathogenicity Analysis

Whole-exome sequencing (WES) was performed by Shanghai We-Health Biomedical Technology Co., Ltd. (Shanghai, China). Genomic DNA samples from sporadic cases and probands and their family members were obtained from peripheral leukocytes using a commercial kit (TIANGEN, Beijing, China). Genomic DNA comprising more than 23,000 genes was subjected to exome sequencing, as previously reported [13]. The raw reads were aligned to the National Center for Biotechnology Information’s (NCBI, https://www.ncbi.nlm.nih.gov/ (accessed on 1 October 2024)) human reference genome (GRCh38/hg19) using the Burrows–Wheeler Aligner version 0.7.10 (Cambridge, UK) and SAMtools version 1.15.1 (Cambridge, UK). The amplified genomic sequences were compared with the *COL2A1* reference sequence NM_001844.5.

The candidate causal gene variants discovered were confirmed using Sanger sequencing, as previously reported [13]. Polymerase chain reaction (PCR) primers were designed using Primer3Plus 6.0 (https://www.primer3plus.com/ (accessed on 3 September 2024)).

The NCBI, 1000 Genome Database (https://www.internationalgenome.org/ (accessed on 15 August 2024)), ExAC (http://exac.broadinstitute.org/ (accessed on 15 August 2024)), and Exome Sequencing Project (https://evs.gs.washington.edu/EVS/ (accessed on 15 August 2024)) were searched for each variant to determine whether it had been previously reported. Population frequencies of these variants were determined using the gnomAD browser (http://gnomad-sg.org/variant (accessed on 15 August 2024)). To predict the pathogenicity of these variants, we performed bioinformatics analysis using tools, including REVEL, SIFT, Polyphen2, MutationTaster, GREP+, LRT, ClinPred, SPIDEX, and SpliceAI, as previously reported [13]. All variants were evaluated according to the standards and guidelines provided by the American College of Medical Genetics and Genomics (ACMG) [14].

Construction of the minigene and transcription analysis were performed to clarify the pathogenicity of *COL2A1*: c.3003+2dup. (1) Wild-type and mutant plasmids: it was constructed based on the mutation information and the patient’s genomic DNA. The cloning vector used was pMini-Cop-GFP, and the cloning site was BamHI/XhoI (Figure S2). Details of the primers used are provided in Figure S2. Wild-type and mutant target fragments were obtained and inserted into the cloning vector, and recombinant plasmids were selected using PCR amplification and Sanger sequencing, as previously reported [15]. (2) Transfection of recombinant vectors expressing *COL2A1*: wild-type and mutated recombinant vectors were transiently transfected into 293T cells using a liposomal transfection reagent (40802ES02, Yeasen, Gaithersburg, MD, USA). The samples were collected after 48 h of incubation. (3) Reverse transcription polymerase chain reaction (RT-PCR) and Sanger sequencing: transfected cells were collected to extract RNA reverse-transcribed cDNA, and gel electrophoresis was performed. PCR amplification products were subjected to Sanger sequencing.

### 2.4. Data Analysis and Statistics

Statistical analyses were performed using Microsoft Excel 2021 (Microsoft Corp, Redmond, WA, USA) and STATA software version 17.0 (StataCorp, LLC, College Station, TX, USA). Two-sided *p* values of <0.05 were considered statistically significant, and normality was tested using the Kolmogorov–Smirnov tests. AL and BCVA were compared using Student’s *t*-test or the Kruskal–Wallis test, respectively. IOL power calculation formulae were compared using the analysis of variance (ANOVA) test.

## 3. Results

### 3.1. Basic Characteristics

Genetic testing was performed in 15 patients with suspected STL1: 11 (73.3%) tested positive for mutations in the STL1 causative gene, COL2A1. Nine gene variants, including four novel and five reported [16,17,18,19,20] mutations, were identified (Table 1). A total of 11 probands and sporadic cases (21 eyes) were enrolled in this study (Table S1), including 11 eyes secondary to RRD that underwent PPV at our hospital and 10 contralateral eyes (Patient 3 had dense corneal opacity in the right eye after a previous vitrectomy that precluded examination). Among 11 STL1 cases, 7 (63.6%) were in men, and 4 were in women; the mean age at onset was 11.5 ± 6.9 (5–30) years. Two eyes had undergone previous surgical procedures (Patients 6 and 4; Table 2). Patient 6 had a scleral buckle for the first RRD at another hospital. Patient 4 had a scleral buckle and PPV combined with lensectomy for the first and second RRD 10 years ago, and this was her third RRD. Seven probands (63.6%) reported a relevant family history; five (45.5%) had high myopia, and two (18.2%) had a history of RRD in their parents.

### 3.2. Genetic Characteristics and Diagnosis

**Figure 1:**
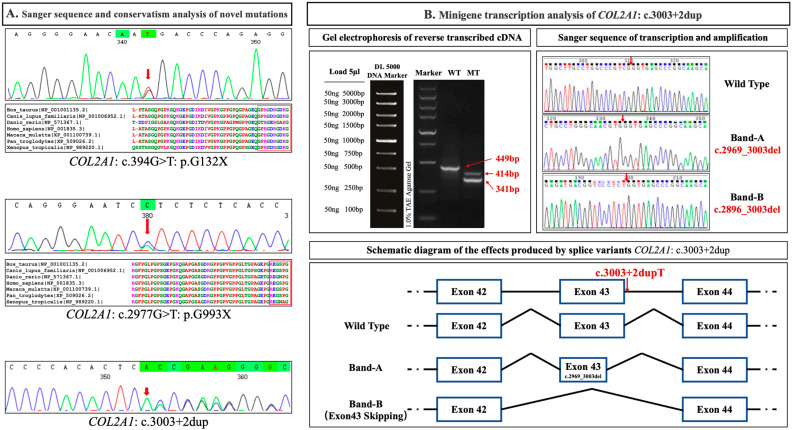
(**A**) Sanger sequencing of novel mutations. (**B**) Results of functional validation experiments using the minigene in the VUS variant: *COL2A1*: NM_001844.5: c.3003+2dup. The red arrows correspond to the sites of nucleotide mutations in both (**A**) and (**B**).

A total of nine variants of COL2A1 were detected and identified (Table 1). Apart from two splice mutations, one frameshift mutation, and two missense mutations, most cases carried nonsense mutations (6/11, 54.5%). Four variants were novel (COL2A1: c.394G>T, p.G132X; c.2977G>T, p.G993X; c.3003+2dup; and c.3853G>C, p.D1285H) (Figure 1A). According to the ACMG guidelines, 77.8% (7/9) of the variants were classified as “pathogenic (P)” or “likely pathogenic (LP)”.

Functional validation experiments on the minigene demonstrated that the VUS variant (COL2A1: c.3003+2dup) produced a new splicing mode during gene transcription. The band sizes of PCR amplification products and Sanger sequencing results verified two aberrant mRNA splicing mutants owing to this mutation, of which band A was of 414 base pairs (bp), band B of 341 bp, and the wild type of 449 bp (Figure 1B). Band A showed a partial deletion of exon 43, whereas band B showed the complete deletion of exon 43, further illustrating impaired gene function due to this nonclassical splicing mutation. Based on minigene results, the pathogenicity class of COL2A1: c.3003+2dup was adjusted from VUS to LP by adding pathogenic evidence of PS3, according to the ACMG guidelines.

### 3.3. Clinical Features

**Figure 2:**
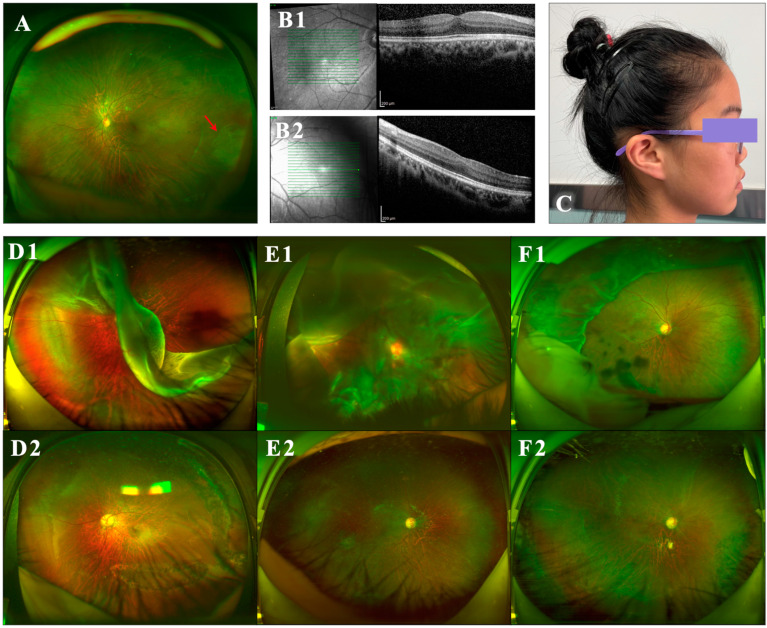
Clinical manifestations in type I Stickler syndrome. (**A**) Vitreous changes in lattice degeneration (the red arrow) in the peripheral retina of an eye without rhegmatogenous retinal detachment (RRD). (**B1**) Typical grade 1 foveal dysplasia on optical coherence tomography (OCT). (**B2**) Typical grade 2 foveal dysplasia on OCT. (**C**) Flat midface. (**D1**–**F1**) Preoperative wide-field fundoscopy of RRD eyes. (**D1**) Left eye of Patient 3. (**E1**) Right eye of Patient 1. (**F1**) Right eye of Patient 6 with holes in the degeneration zone. The patient experienced unsuccessful scleral buckling at another hospital. (**D2**–**F2**) Wide-field fundoscopy of the same eye 3 months after silicone oil removal (SOR), respectively, in (**D1**–**F1**). (**D2**) Patient 3. (**E2**) Patient 1. (**F2**) Patient 6. The scleral buckle was removed before vitrectomy.

In the eyes with RRD, the interval between the onset of symptoms and surgery ranged from 1 to 8 weeks, with a mean ± standard deviation (SD) of 3.4 ± 2.1 weeks. At the first visit, 90.9% of eyes (10/11) were macula-off, 63.6% (7/11) had grade C proliferative vitreoretinopathy (PVR), and 36.4% (4/11) presented with giant holes, including ciliary dissection (Figure 2). Seven eyes (63.6%) had preoperative BCVA worse than 20/400 (six eyes hand move and one eye 20/1000), 18.2% eyes between 20/400 and 20/70, and 18.2% eyes ≥ 20/70. Preoperative BCVA of RRD eyes was significantly poorer than that of contralateral eyes (logMAR, middle [range], 0.82 [0.15–1.70] vs. 0.10 [0–0.30], *p* = 0.0120). The mean AL of RRD eyes was 27.35 ± 2.76 mm, which was not significantly different from contralateral eyes without RRD (27.18 ± 1.15 mm) (*p* = 0.8588). IOP was 9.7 ± 3.1 (6.1–14) vs. 16.2 ± 4.6 (11–24) mmHg between eyes with and without RRD (*p* = 0.0021). FH was observed in 60% of the contralateral eyes (6/10; Table 3), of which 50% was typical grade 1 and 10% was typical grade 2 (Figure 2).

In addition to ocular manifestations, the proportions of systemic abnormalities of STL1 were 100% flat midface (Figure 2), 54.5% (6/11) depressed nasal bridge, 27.3% (3/11) sensorineural deafness, 27.3% (3/11) osteoarthritis, and 9.1% (1/11) cleft palate (Table 3).

### 3.4. Treatment Procedures

All patients accepted PPV as the first surgery, in which ten underwent silicone oil tamponade, and one underwent C_3_F_8_ tamponade. Only one eye (9.1%) underwent vitrectomy combined with cataract removal. During the follow-up, 81.8% (9/11) of the cases underwent additional surgical procedures, including SOR, cataract extraction, IOL implantation, or PPV for recurrent RRD. The reasons for the first additional surgery included SOR with cataract extraction and IOL implantation (n = 4, 36.4%), SOR (n = 4, 36.4%), and PPV for recurrent RRD (n = 1, 9.1%). Two eyes (18.2%) underwent a second procedure for cataract extraction, IOL implantation, and SOR with cataract extraction. The mean interval between the first PPV and final SOR was 7.11 (3–17) months. With an average of 2.0 (1–3) surgical procedures, only one eye (9.1%) failed to achieve the final retinal reattachment. Considering the risk of RRD in fellow eyes with STL1, lattice degeneration in 10 contralateral eyes was treated with retinal laser retinopexy, and all eyes were free of retinal detachment or other secondary changes during the follow-up.

### 3.5. Surgery Outcomes and Complications

After no less than 18 months (mean ± SD = 32.9 ± 25.7; range, 18–96) of follow-up, the final retinal reattachment rate was 90.9%. At the last follow-up, BCVA significantly improved in 81.8% of the eyes, with a middle logMAR BCVA of 0.52 (Snellen 20/66, *p* = 0.0148).

High IOP (mmHg, range, 23.4–30.7) occurred in nine eyes (81.8%); it occurred in all cases within 3 months postoperatively and was controlled with anti-glaucoma medications. Eight eyes (72.7%) had complicated cataracts. Two eyes (18.2%) developed recurrent RRD, one of which underwent retinal reattachment after the second PPV. Another patient (Patient 11) developed recurrent RRD after PPV and silicone oil tamponade. During follow-up, the RRD eye showed a persistently low IOP and developed silicone oil dependence. Considering the poor visual prognosis, the patient discontinued further PPV (requiring silicone oil tamponade). After 2 years of postoperative follow-up, band keratopathy and significant silicone oil emulsification developed and persisted until the final follow-up (half a year prior). The IOP remained at approximately 11 mmHg.

### 3.6. IOL Power Calculation Comparison

**Figure 3:**
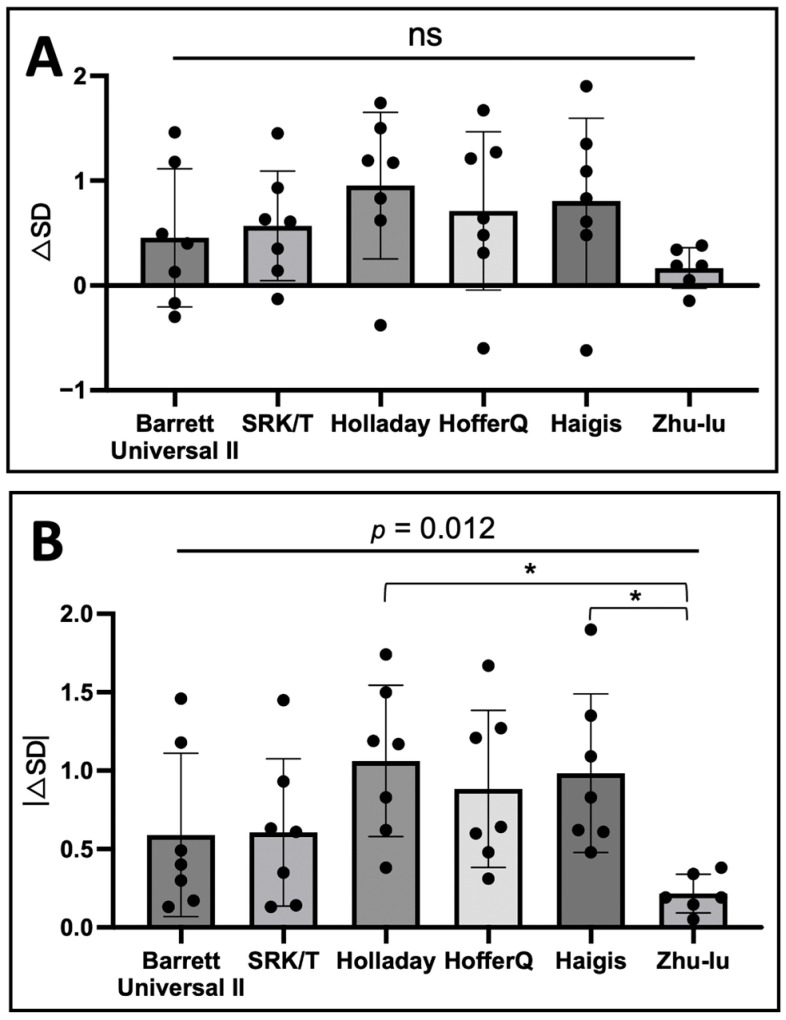
(**A**) The mean prediction error (△SD) of intraocular lens power between different formulae. (**B**) The mean absolute prediction error (|△SD|) of intraocular lens power between different formulae. * *p <* 0.0017.

△SD was not significant between formulae (*p* = 0.176), and |△SD| was significant between formulae (*p* = 0.012; Figure 3). Post hoc analysis revealed significant differences between Holladay and Zhu-Lu and between Haigis and Zhu-Lu (*p* = 0.0017).

## 4. Discussion

In this study, we performed a retrospective analysis and identified nine pathogenic variants of *COL2A1* associated with STL1. Among these, four (44.4%) were novel, including *COL2A1*: c.394G>T, p.G132X; c.2977G>T, p.G993X; c.3003+2dup; and c.3853G>C, p.D1285H. We also observed promising PPV outcomes in patients with RRD secondary to STL1. With a mean of 32.9 months of follow-up, 90.9% of the eyes with RRD achieved reattachment, and 81.8% of the eyes had improved BCVA.

Comprehensive bioinformatics and pathogenicity predictions were performed for every variant to clarify the relationship between mutations and the disease. Further functional validation experiments on the VUS variant (*COL2A1*: c.3003+2dup) provided additional evidence that the mutation impaired the gene function by producing a new splicing mode during transcription. This demonstrates the diagnostic value of functional validation experiments for determining pathogenicity, especially for novel mutations lacking previous reports and de novo mutations lacking evidence of lineage co-segregation. In the remaining suspected cases, WES was unable to identify any pathogenic mutations; whole-genome sequencing would be another way to explore these undiagnosed cases.

Stickler et al. [4] described the manifestations of STL1, including myopia, RRD, cataract, glaucoma, flat midface, cleft palate, small jaw, hearing loss, skeletal manifestations, and joint problems. In our study, only 27.3% of the patients had hearing loss, and 27.3% had skeletal disease, whereas the others only manifested facial changes. When a child presents with RRD and high myopia, it is important to inquire about the family history and assess potential systemic symptoms. If STL1 is suspected, genetic testing would be helpful for diagnosis and the timely initiation of multidisciplinary follow-up.

The anatomical or visual outcomes of STL after surgery for RRD have varied in previous studies (Table 4). Burdová et al. [21] achieved 77% eye reattachment, with an average of 2.3 surgical procedures. Corcóstegui et al. [22] achieved 100% eye reattachment, in which 87.5% of the eyes underwent scleral buckling combined with PPV. Reddy et al. [2] achieved 100% retinal reattachment after a mean of 3.1 surgical procedures, but 63% of the eyes were still treated with silicone oil tamponade at the last visit. A study from Saudi Arabia [23] achieved 70.8% primary reattachment of the retina, and BCVA did not improve in 37.7% of eyes after surgery. To the best of our knowledge, there have been no previous reports specifically on the surgical prognosis of RRD secondary to STL1—our study is the first to focus on it.

We achieved 90.9% anatomical reattachment after a mean of two surgical procedures, and 81.8% of eyes showed improved BCVA after surgery, with a middle logMAR BCVA of 0.52 (0.15–0.82). The reasons we obtained satisfactory outcomes may be as follows. (a) We used a 23- or 25-gauge minimally invasive surgery system for all patients, and a valved cannula was helpful in stabilizing intraocular perfusion. (b) Repeated cortex removal using TA helped completely clean the vitreous cortex, especially when posterior precortical vitreous pockets may exist [24]. (c) Less severe initial manifestations. In one study, 92.9% of eyes with macular involvement, 62.9% with total retinal detachment, and 47.1% (9.1% in our study) with complicated cataracts were reported at the initial visit [23]. In another study, 66.7% with PVR (13.3% inoperable) [21] and 50% with PVR (7.1% inoperable) [23] were documented (no inoperable eyes were included in our study). These factors may have contributed to poorer outcomes in previous reports.

PPV were conducted in all cases, although there were more options, including scleral buckling with cryopexy or combined PPV with scleral buckling. However, there is no definite evidence as to which method is the best choice. Corcóstegui et al. [22] achieved 100% retinal reattachment using scleral buckling with PPV. Combined surgery seems to be a more appropriate method; however, long-term complications of scleral buckling need to be considered [25,26].

Since retinal detachment surgery in STL1 is challenging and the prognosis is poor, prophylactic interventions have been recommended by many retinal specialists, especially in patients with a history of contralateral RRD. Cryotherapy or argon laser photocoagulation has been used [27,28,29,30]. Although there is no consensus or guideline for prophylactic RRD treatment in STL1, previous studies have shown favorable outcomes [27,28,29,30]. All contralateral eyes of our patients underwent laser photocoagulation and remained stable during follow-up.

At the final visit, 72.7% (8/11) of the eyes were aphakia or pseudophakia in our study, of which seven had complicated cataracts after surgery, and one had high myopia of −12 Diopter before surgery who asked to remove the lens in first PPV. For patients undergoing IOL implantation, IOL power calculation is essential for obtaining good visual quality. In our study, the Zhu-Lu formula showed better accuracy for IOL power prediction than other formulas. However, this result was limited by the small sample size of our study.

There were still some limitations of this study. First, all the participants included in this study underwent simple PPV. There were no patients who underwent other surgical procedures such as scleral buckle or scleral buckle combining PPV. Thus, it was difficult to compare different surgical procedures. Second, the study had a relatively small sample size and follow-up duration was not long enough for these young patients. Integrating our data with patient information from other ophthalmic centers would be an option to provide more generalized evidence for this rare disease.

## 5. Conclusions

By combining clinical manifestations and genetic testing, STL1 could be precisely diagnosed. In this study, PPV showed fine anatomical and functional results for RRD secondary to STL1. With proper surgical treatment, RRD secondary to STL1 can achieve anatomical reattachment and improve visual quality. It is important to pay attention to both eyes with STL1, and prophylactic treatment should be considered when needed.

## Figures and Tables

**Table 1:** Bioinformatic analysis of *COL2A1* gene mutations in sporadic cases and probands with type I Stickler syndrome included in this study.

No.	Type	Exon	Nucleotide Changes	Protein Changes	Effect	Origin	Reference	Mutation Taster	REVEL	GERP+	SpliceAI	gnomAD_ Exome _ALL	ACMG
1	het	exon 6	c.394G>T	p.G132X	nonsense	N/A	Novel	DC (1)	-	Conserved (5.09)	0	-	P
2	het	exon 43	c.2977G>T	p.G993X	nonsense	father	Novel	DC (1)	-	Conserved (5.44)	DG (0.28) *	0.00000654	P
3	het	intron 43	c.3003+2dup	-	splice	father	Novel	-	-	-	-	-	LP
4	het	intron 12	c.817-9G>A	-	splice	father	reported [16]	DC_A (1)	-	Conserved (3.88)	AG (0.92) AL (0.78) *	-	LP
5	het	exon 17	c.1030C>T	p.R344X	nonsense	mother	reported [17]	DC_A (1)	-	Conserved (3.35)	0	-	P
6	het	exon 25	c.1658_ 1675dup	p.E553_ G558dup	frameshift	father	reported [18]	DA (1)	-	-	-	-	LP
7	het	exon 44	c.3106C>T	p.R1036X	nonsense	de novo	reported [19]	DC_A (1)	-	Conserved (4.48)	DG (0.02)	0.000004	P
8	het	exon 44	c.3106C>T	p.R1036X	nonsense	de novo	reported [19]	DC_A (1)	-	Conserved (4.48)	DG (0.02)	0.000004	P
9	het	exon 17	c.1030C>T	p.R344X	nonsense	mother	reported [17]	DC_A (1)	-	Conserved (3.35)	0	-	P
10	het	exon 51	c.3853G>C	p.D1285H	missense	de novo	Novel	DC (1)	D (0.877)	Conserved (4.62)	0	-	VUS
11	het	exon 26	c.1693C>T	p.R565C	missense	mother	reported [20]	DC_A (1)	LD (0.687)	Conserved (5.05)	0	-	P

*COL2A1* gene reference sequence: NM_001844.5. Abbreviations: N/A, unavailable; het, heterozygous; DC_A, disease causing_automatic; DC, disease causing; D, damaging; LD, likely damaging; DG, donor gain; AG, acceptor gain; AL, acceptor loss; P, pathogenic; LP, likely pathogenic; VUS, variants of uncertain significance; *, Values of SpliceAI score ≥ 0.2 indicate deleterious effects on mRNA splicing.

**Table 2:** Basic characteristics of patients with type I Stickler syndrome.

Characteristics	No. of Eyes (%)
RRD eyes (left/right)	11 (3/8) (52.4)
Contralateral eyes (left/right)	10 (8/2) (47.6)
Surgical history of RRD eyes	
Scleral buckle	2 (18.2)
PPV combined lensectomy	1 (9.1)
Family history	
High myopia	5 (45.5)
RRD	2 (18.2)

Abbreviations: RRD, rhegmatogenous retinal detachment; PPV, pars plana vitrectomy.

**Table 3:** Clinical features of RRD eyes and contralateral eyes and systemic manifestations in patients with type I Stickler syndrome.

Features	RRD Eyes	Contralateral Eyes	*p* Value
AL (mm), mean ± SD (range)	27.35 ± 2.76 (23.57–31.19)	27.18 ± 1.15 (25.02–28.74)	0.8588
IOP (mmHg), mean ± SD (range)	9.7 ± 3.1 (6.1–14)	16.2 ± 4.6 (11–24)	0.0021
BCVA (logMAR), middle (range)	0.82 (0.15–1.70)	0.10 (0–0.30)	0.0120
PVR			
A	0		
B	4 (36.4%)		
CA	0		
CP	5 (45.5%)		
CA + CP	2 (18.2%)		
Macular condition			
Macula-off	10 (90.9%)		
Typical grade 1 FH		5 (50%)	
Typical grade 2 FH		1 (10%)	
Types of retinal hole/tear			
Hole in degeneration zone	4 (36.4%)		
Horse-shoe tear	2 (18.2%)		
Giant hole	5 (45.5%)		
Orofacial, Skeletal, Auditory abnormalities			
Flat midface	11(100%)		
Depressed nasal bridge	6 (54.5%)		
Cleft palate	1 (9.1%)		
Osteoarthritis	3 (27.3%)		
Sensorineural deafness	3(27.3%)		

Abbreviations: RRD, rhegmatogenous retinal detachment; AL, axial length; SD, standard deviation; IOP, intraocular pressure; BCVA, best corrected visual acuity; PVR, proliferative vitreoretinopathy; FH, foveal hypoplasia.

**Table 4:** Summary of previous research on RRD surgery in patients with type I Stickler syndrome.

Authors	Study Characteristics	Intervention	Follow-Up Time	Reattachment Rate	Surgery Counts	PVR (%)	VA Outcome	Complications
Reddy et al., 2016, United States [2]	Retrospective case series, 16 eyes from 13 patients	SB, PPV, or SB combining PPV	94 months (5–313) months	100%	3.1 (1–13)	N/A	31% improved 31% unchanged 37.5% declined	75% PVR 63% silicone oil dependent
Burdová et al., 2023, Czech Republic [21]	Retrospective case series, 15 eyes from 9 patients	SB, Cryopexy, or SB combining PPV	9.6 years (5–16) years	77%	2.3 (1–6)	66.7	23.1% < 20/400 15.4% 20/400–20/70 61.5% ≥ 20/70 improved: unclear	76.9% PVR
Corcóstegui et al., 2024, Spain [22]	Retrospective case series, 24 eyes from 18 patients	87.5% SB combining PPV 12.5% SB	10.2 years (3–33) years	100%	1.21 (1–6)	8.3	8.3% < 20/400 16.7% 20/400–20/70 75% ≥ 20/70 improved: 20/252 to 20/63	16.7% PVR 50% cataract
Alshahrani et al., 2016, Saudi Arabia [23]	Retrospective case series, 70 eyes from 62 patients	15.4% SB, 10.8% PPV, 73.8% SB combining PPV	3.8 years (1 month–14 years)	93.8%	N/A	50	62.3% improved 28.6% unchanged 7.1% declined	27.1% raised IOP 27.1% recurrent RD 25.7% cataract 14.3% PVR 8.57% band keratopathy 7.14% ERM 1.4% choroidal hemorrhage

Abbreviations: VA, visual acuity; SB, scleral buckle; PPV, pars plana vitrectomy; PVR, proliferative vitreoretinopathy; N/A, unavailable; IOP, intraocular pressure; RD, retinal detachment; ERM, epiretinal membrane; RRD, rhegmatogenous retinal detachment.

## Data Availability

Data are available from the authors upon reasonable request.

## Supplementary Materials

The following supporting information can be downloaded at https://www.mdpi.com/article/10.3390/genes15111455/s1: Figure S1: Pedigree of Patient 11 and Patient 4. I-1 (A) and I-2 (B) underwent rhegmatogenous retinal detachment surgery in another hospital and underwent complicated cataract surgery at our hospital. They were recruited for intraocular lens power calculation formula comparison; Figure S2: Detailed information on minigene and transcription analysis experiments, including the structure of minigene cloning vectors and the primers used in minigene construction, amplification product identification, and RT-PCR. RT-PCR, reverse transcription-polymerase chain reaction; Table S1: Baseline characteristics of Type 1 Stickler syndrome patients with rhegmatogenous retinal detachment.
