# Supplementary material for: Rhegmatogenous Retinal Detachment Secondary to Type I Stickler Syndrome: Diagnosis, Treatment and Long-Term Outcomes
Source: Genes (Basel). 2024 Nov 11;15(11):1455. doi: 10.3390/genes15111455 (PMC11594037; doi:10.3390/genes15111455)
Supplement: Supplementary file 1 [file genes-15-01455-s001.zip › Supplementary_Materials.pdf]

Supplementary Materials

Table S1: Baseline characteristics of Type 1 Stickler Syndrome patients with rhegmatogenous retinal detachment

No.	Sex/ Onset Age/Y	Eye	RRD	Macula/PVR	Retinal hole/tear	FH	Orofacial abnormalities	Skeletal and auditory abnormalities
1	F/6	OD	Y	off/CP	Giant hole	-	Flat midface, Depressed nasal bridge, Cleft palate	Osteoarthritis Sensorineural deafness Hearing aid implants
		OS	N	-	-	Without FH		
2	M/7	OD	Y	off/CA+CP	Giant hole	-	Flat midface, Depressed nasal bridge	-
		OS	N	-	-	Grade 1 FH		
3	M/10	OD	Y	N/A	N/A	-	Flat midface, Depressed nasal bridge	Osteoarthritis Sensorineural deafness Hearing aid implants
		OS	Y	off/CP	Giant hole with ciliary dissection	-		
4	F/30	OD	N	-	-	Grade 1 FH	Flat midface	-
		OS	Y	off/CP	Hole in degeneration	-		
5	M/12	OD	Y	off/B	Hole in degeneration	-	Flat midface	-
		OS	N	-	-	Without FH		
6	M/15	OD	Y	off/CP	Hole in degeneration	-	Flat midface, Depressed nasal bridge	Mild sensorineural deafness
		OS	N	-	-	Grade 1 FH		
7	M/8	OD	N	-	-	Grade 2 FH	Flat midface	-
		OS	Y	off/CP	Giant hole	-		
8	F/8	OD	Y	off/B	Hole in degeneration	-	Flat midface, Depressed nasal bridge	-
		OS	N	-	-	Grade 1 FH		

9	M/5	OD	Y	off/B	Horse-shoe tear	-	Flat midface, Depressed nasal bridge	-
		OS	N	-	-	Grade 1 FH		
10	M/13	OD	Y	on/B	Horse-shoe tear	-	Flat midface	Osteoarthritis
		OS	N	-	-	Without FH		
11	F/12	OD	Y	off/CP	Giant hole	-	Flat midface	-
		OS	N	-	-	Without FH		

Abbreviations: AL, axial length; PVR, proliferative vitreoretinopathy; BCVA, Best corrected visual acuity; PPV, pars plana vitrectomy; SiO, silicone oil tamponade; FH, foveal hypoplasia; CA, CP, OD, OS, M, male; F, female; Y, yes; N, no.

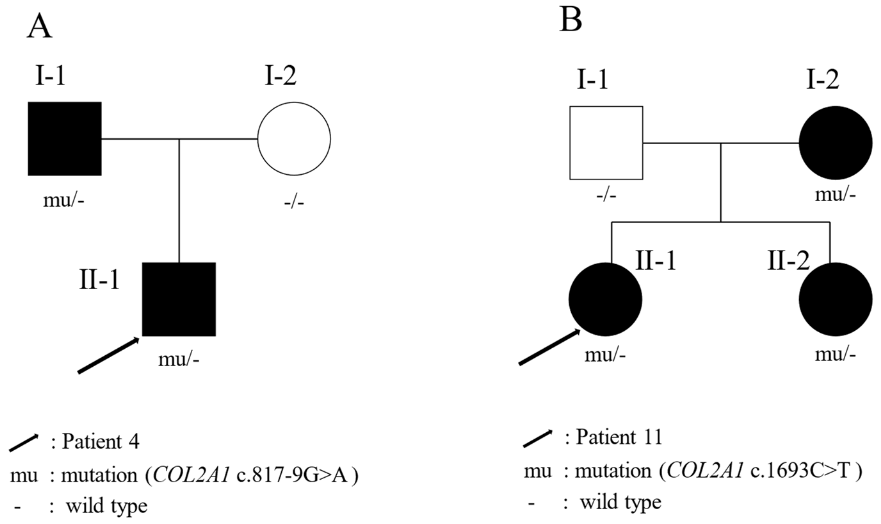

Figure S1: Pedigree of Patient 11 and Patient 4. I-1(A) and I-2 (B) underwent rhegmatogenous retinal detachment surgery in another hospital and underwent complicated cataract surgery at our hospital. They were recruited for intraocular lens power calculation formula comparison.

● Structure of MINI-gene cloning vectors

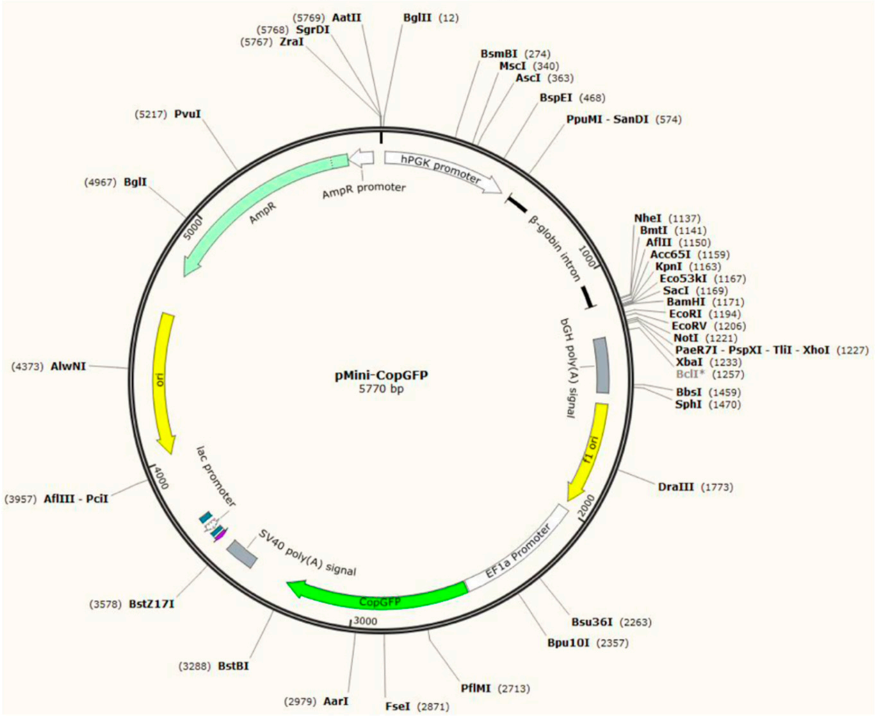

● The primers used in MINI-gene construction

Name	Primer Sequence (5'→3')
COL2A1-F	AAGCTTGGTACCGAGCTCGGATCCG<br>GCAACCCTGGACCCCCTGGTCCCCC
COL2A1-R	TTAAACGGGCCCTCTAGACTCGAGC<br>TCTCGTCCAGGTTCACCTGCAGGAC

● The primers used in amplification product identification

Name	Primer Sequence (5'→3')
β-globin intron-F	GATATACACTGTTTGAGATGAGGA
BGH-R	TAGAAGGCACAGTCGAGG

● The primers used in RT-PCR

Name	Primer Sequence (5'→3')
MiniRT-F	GGCTAACTAGAGAACCCACTGCTTA
COL2A1-RT-R	CTCTCGTCCAGGTTCACCTGCAGG

Figure S2. Detailed information on MINI-gene and transcription analysis experiments, including the structure of MINI-gene cloning vectors and the primers used in MINI-gene construction, amplification product identification, and RT-PCR. RT-PCR, reverse transcription-polymerase chain reaction
